# Deletion of the Gamma Subunit of ENaC in Endothelial Cells Does Not Protect against Renal Ischemia Reperfusion Injury

**DOI:** 10.3390/ijms222010914

**Published:** 2021-10-09

**Authors:** Stephanie M. Mutchler, Mahpara Hasan, Donald E. Kohan, Thomas R. Kleyman, Roderick J. Tan

**Affiliations:** 1 Department of Medicine, University of Pittsburgh, Pittsburgh, PA 15261, USA; smm226@pitt.edu (S.M.M.); tanr@pitt.edu (R.J.T.); 2 Department of Biological Sciences, Carnegie Mellon University, Pittsburgh, PA 15213, USA; mhasan1@pennstatehealth.psu.edu; 3 Department of Medicine, University of Utah, Salt Lake City, UT 84112, USA; donald.kohan@hsc.utah.edu; 4 Departments of Medicine, Cell Biology, and Pharmacology and Chemical Biology, University of Pittsburgh, Pittsburgh, PA 15261, USA

**Keywords:** ENaC, ischemia reperfusion injury, nitric oxide

## Abstract

Acute kidney injury due to renal ischemia-reperfusion injury (IRI) may lead to chronic or end stage kidney disease. A greater understanding of the cellular mechanisms underlying IRI are required to develop therapeutic options aimed at limiting or reversing damage from IRI. Prior work has shown that deletion of the α subunit of the epithelial Na+ channel (ENaC) in endothelial cells protects from IRI by increasing the availability of nitric oxide. While canonical ENaCs consist of an α, β, and γ subunit, there is evidence of non-canonical ENaC expression in endothelial cells involving the α subunit. We therefore tested whether the deletion of the γ subunit of ENaC also protects mice from IRI to differentiate between these channel configurations. Mice with endothelial-specific deletion of the γ subunit and control littermates were subjected to unilateral renal artery occlusion followed by 48 h of reperfusion. No significant difference was noted in injury between the two groups as assessed by serum creatinine and blood urea nitrogen, levels of specific kidney injury markers, and histological examination. While deletion of the γ subunit did not alter infiltration of immune cells or cytokine message, it was associated with an increase in levels of total and phosphorylated endothelial nitric oxide synthase (eNOS) in the injured kidneys. Our studies demonstrate that even though deletion of the γ subunit of ENaC may allow for greater activation of eNOS, this is not sufficient to prevent IRI, suggesting the protective effects of α subunit deletion may be due, in part, to other mechanisms.

## 1. Introduction

Renal ischemia-reperfusion injury (IRI) is a clinical complication that can lead to acute kidney injury (AKI) and progression to chronic kidney disease (CKD) or end-stage kidney disease. IRI is a common contributor to AKI due to sepsis, shock, or trauma and is also observed in kidney transplants after harvest [1]. Treatment options for IRI-associated AKI are largely supportive in nature, including maintenance of kidney perfusion during shock and the use of acute dialysis when necessary. A better understanding of the mechanisms of IRI is required to develop more targeted treatments to ameliorate kidney damage and prevent disease progression.

IRI consists of two distinct phases. The first phase, ischemia, is characterized by low blood flow and poor tissue oxygenation. While the cortex receives 100% of the blood entering the kidney, the medulla receives only a fraction of the total perfusion even under normal conditions, making the outer medulla especially prone to ischemic injury [2]. Blood flow can be reduced by up to half in this segment during ischemia, and this creates an oxygen debt even after restoration of blood flow [2]. This lack of oxygen results in mitochondrial dysfunction and reduced ATP generation, leading to cellular acidosis and intracellular calcium accumulation in association with reduced activities of the sodium-potassium-ATPase and ATP-dependent calcium pump [3]. The second phase of IRI occurs during reperfusion. While restoration of blood flow is necessary for overall tissue survival, the rapid influx of oxygen is associated with a burst of reactive oxygen species that can directly damage DNA, proteins, and lipids, alter membrane structure and mitochondrial integrity, and activate or de-activate signaling pathways leading to cell death of tubular epithelia [3]. Reductions in renal blood flow can also persist, contributing to an “extension phase” in which injury continues even after reperfusion. This phenomenon, which appears to target the outer medulla, is likely due to congestion, edema, inflammation and vasoconstriction [4]. Proper reoxygenation during reperfusion requires dilation of the renal vasculature which is accomplished, in part, by the creation of the gaseous vasodilator nitric oxide (NO) [5].

NO has anticoagulant, antiatherogenic, and antioxidant properties, and it has been shown to have protective effects in IRI [6,7,8]. Endothelial nitric oxide synthase (eNOS) is located within endothelial cells of the kidney and produces NO from L-arginine. Enzymatic activity of eNOS is regulated by a multitude of factors [9]. Phosphorylation of the enzyme at Ser1177 (Ser1179 in mice) results in a conformational shift that facilitates the flux of electrons to increase catalytic activity [10]. Shear stress-induced mechanical deformation can cause this phosphorylation and activation of eNOS in endothelial cells through increases in calcium signaling [11,12]. Pathologic stiffening of the endothelial membrane decreases responsiveness to shear stress, ultimately leading to decreased NO production [13].

The epithelial Na+ channel (ENaC, encoded by the *Scnn1a*, *Scnn1b*, and *Scnn1g* genes) has been shown to contribute to endothelial stiffness and subsequently limit NO generation in endothelial cells [14]. ENaC is a trimeric channel canonically consisting of an α, β, and γ subunit. All three subunits possess two transmembrane domains connected by a large extracellular loop that sense changes in the extracellular environment such as fluid shear stress. While best known for modulating sodium and fluid absorption in the epithelium of the lung and kidney, it has more recently been shown to increase local intracellular Na+ concentrations, leading to actin polymerization and subsequent membrane stiffening that limits eNOS activation in endothelial cells [15,16,17].

Recent work found that deletion of the α subunit of ENaC in endothelial cells reduced renal injury after ischemia-reperfusion in mice [18]. This protection was attributed to increased NO availability, as human umbilical vein endothelial cells (HUVECs) treated with the ENaC inhibitor amiloride had greater levels of eNOS dimerization and activation when exposed to hypoxia compared to untreated cells [18]. ENaC are members of the ENaC/degenerin family of ion channels [19]. While all three ENaC subunits are required for a canonical channel, the α subunit has been shown to interact with other members of the ENaC/degenerin family, such as acid sensing ion channel 1a (ASIC1a) to form non-canonical, non-selective cation channels [20]. These non-canonical channels have been shown to play a role in promoting endothelial barrier function in the setting of pneumonia, presumably independent of the role of the α subunit within traditional ENaC channels [21]. Therefore, we tested whether deletion of γ subunit of ENaC in endothelial cells provides similar protection in IRI, as a way to determine whether canonical ENaC channels contribute to IRI-associated AKI or whether the protective effects may be due to non-canonical channels. 

## 2. Results

### 2.1. Generating an Endothelial ENaC γ Subunit Knockout

**Figure 1:**
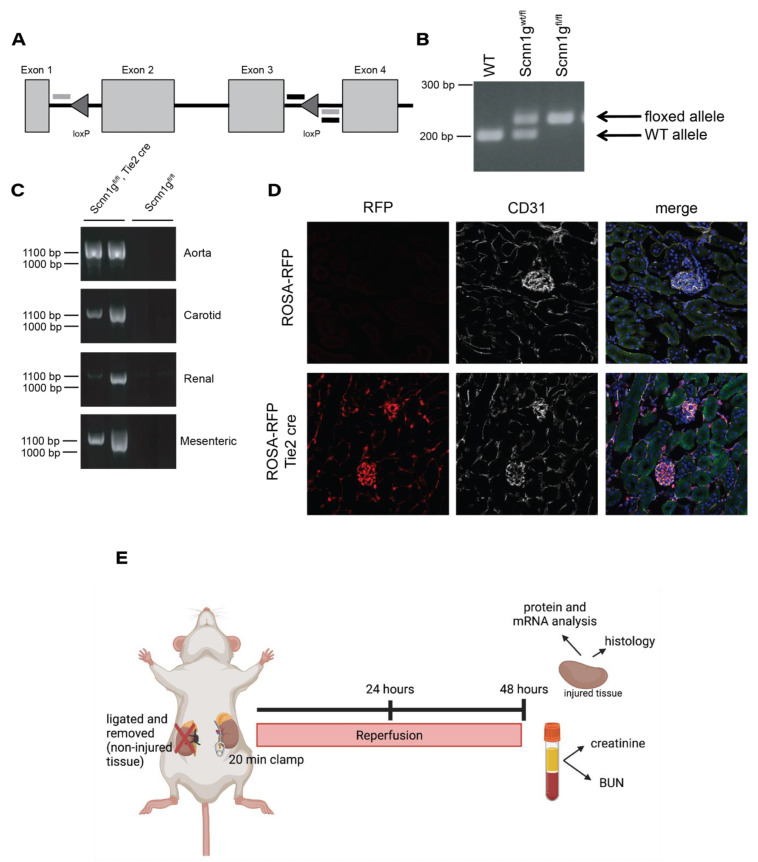
Design and validation of a mouse model with loxP insertions in the γ subunit of ENaC. (**A**) Tissue specific KO of the γ subunit of ENaC was achieved through creation of a mouse containing two loxP sites surrounding the second and third exons of the *Scnn1g* gene. Genotyping primers were designed that flanked the second loxP site (designated by black, horizontal lines) and primers were also developed to validate successful excision (designated by gray horizontal lines). (**B**) Genomic DNA isolated from mouse tail snips were added to PCR reactions containing genotyping primers. WT animals had only a lower ~200 bp whereas insertion of the loxP site was indicated by an upward shift to ~230 bp. Heterozygous animals (Scnn1gwt/fl) displayed both bands while homozygous animals (Scnn1gfl/fl) had only the upper band. (**C**) Aorta, carotid, renal and mesenteric arteries were isolated from Scnn1gfl/fl animals with or without expression of an endothelial specific cre under the Tie2 promoter. DNA was extracted and added to a PCR reaction with excision validation primers. Presence of a product is seen only in cre-expressing animals. (**D**) Kidneys from Tie2 cre animals crossed to a ROSA-RFP reporter mouse denote cre-expressing cells in red. An antibody to the endothelial marker CD31 is shown in white with green autofluorescence marking the tissue outline and blue DAPI staining showing the location of nuclei. (**E**) Schematic of experimental procedure showing that at the time of surgery, the left renal vasculature was clamped for 20 min and the right kidney was ligated and removed. Reperfusion was allowed for 48 h before the animals were sacrificed and downstream analysis performed.

An animal lacking expression of the γ subunit of ENaC specifically in endothelial cells was generated using the cre-lox system. Two loxP sites were inserted in the introns surrounding exons 2 and 3 of the *Scnn1g* gene, located on chromosome 16p12.2 (Figure 1A). PCR primers were designed that surrounded the second loxP site (Figure 1A, designated by horizontal black lines) to facilitate genotyping of the animals. In animals where the loxP site was not present, the PCR product was ~200 bp whereas the insertion was indicated by an upward shift to ~230 bp. Heterozygous animals displayed both bands (Figure 1B). Mice homozygous for *Scnn1g* flanked by loxP sites (floxed (fl)) were crossed to animals homozygous for floxed *Scnn1g* constitutively expressing cre driven by the Tie2 promoter to achieve animals with *Scnn1g* knockout in endothelial cells as well as littermate controls (homozygous for floxed *Scnn1g* but lacking cre). Primers were designed that would only create a PCR product upon excision of exons 2 and 3 to validate the system (Figure 1A, designated by horizontal gray lines). The aorta, carotid, renal, and mesenteric arteries were dissected from animals containing the loxP sites alone (Scnn1gfl/fl) or animals containing both the loxP sites and cre expression (Scnn1gfl/fl, tie2 cre) and DNA isolated. PCR demonstrated that excision only occurred in animals expressing cre (Figure 1C). To validate the specificity of the cre, tie2 cre animals were crossed to a ROSA-RFP reporter animal, and tissues imaged for the overlap of RFP reporter and CD31 as a marker of endothelial cells (Figure 1D). RFP was selectively expressed in CD31-expressing endothelial cells.

### 2.2. Serum and Tissue Indicators of Renal Damage Do Not Differ between Groups after IRI

**Figure 2:**
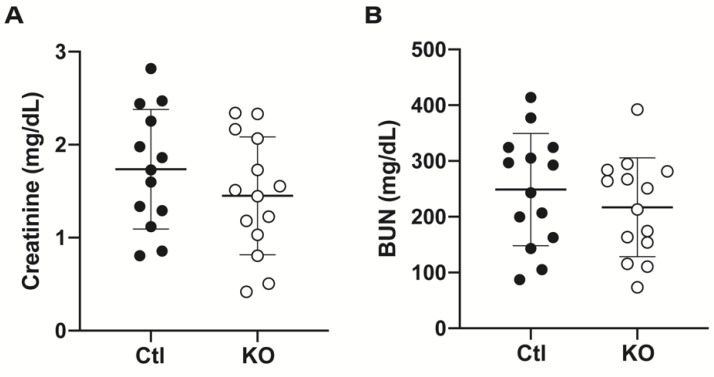
Serum markers of kidney injury do not differ between control animals and animals lacking endothelial expression of the γ subunit of ENaC after IRI. Serum collected from control and knockout animals after 48 h of reperfusion showed no significant difference in levels of creatinine (**A**) or blood urea nitrogen (**B**). n = 13 to 14 for each measure; data represented as mean ± SD; unpaired t-tests performed to determine existence of difference with definition of statistical significance set at *p* < 0.05.

Experimental procedures are outlined in Figure 1E. Both homozygous floxed animals (control (Ctl)) and cre-expressing floxed animals (knockout (KO)) were subjected to 20 min of unilateral renal ischemia. The contralateral kidney was simultaneously excised and used as “uninjured” tissue. After 48 h of reperfusion, blood and tissue were collected. Serum levels of creatinine and urea (blood urea nitrogen (BUN)) were used as indicators of glomerular damage, as an increase in the circulating levels suggests decreased glomerular filtration rate. Control and knockout animals showed similar levels of creatinine (1.7 ± 0.6 mg/dL vs. 1.5 ± 0.6 mg/dL) (Figure 2A) and BUN (248.8 ± 100.8 mg/dL vs. 217.1 ± 88.7 mg/dL) (Figure 2B), suggesting no difference in degree of glomerular injury or filtration ability.

**Figure 3:**
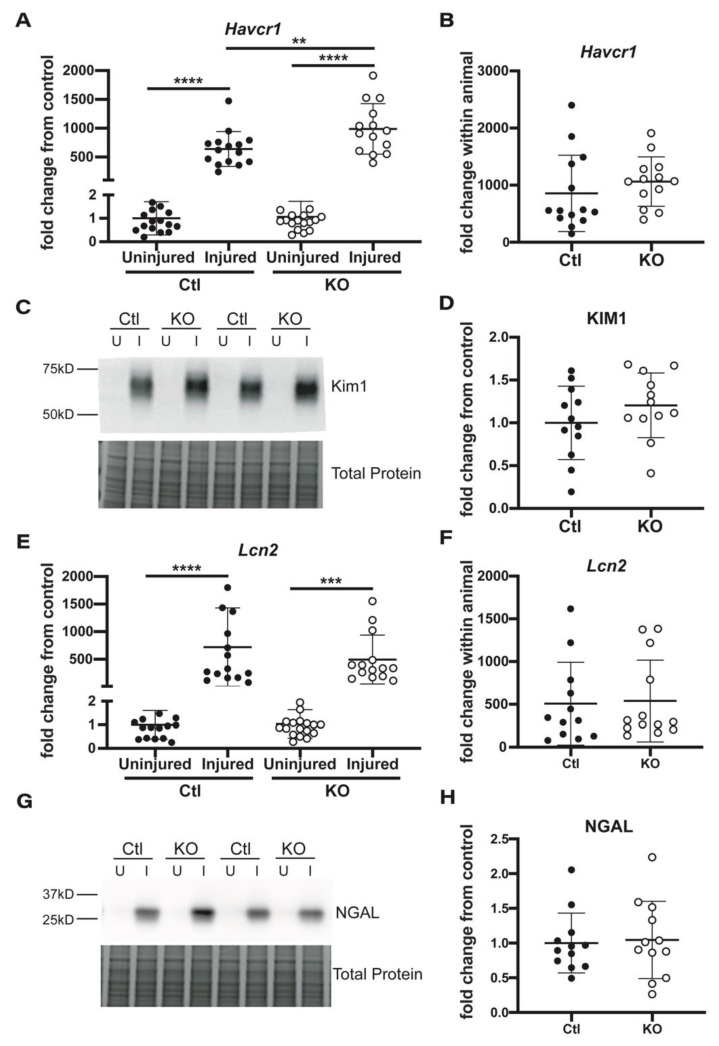
KIM-1 and NGAL expression in kidneys from mice subjected to IRI. (**A**) qPCR was performed to analyze the expression of *Havcr1* message, the gene which encodes for KIM-1. Data were analyzed by the 2^−^^ΔΔ^^Ct^ method to determine fold change from the control, uninjured kidney. (**B**) These same data were analyzed in each individual mouse to determine *Havcr1* expression in the kidney after injury, relative to the uninjured kidney. (**C**) Western blot was used to measure protein levels of KIM-1, with the representative blot illustrating noticeable expression in only the injured (I) kidneys of both control and knockout animals as compared to their uninjured (U) counterpart. Bands of interest were normalized to total protein as determined by a simultaneously run Coomassie stained gel, with a representative portion shown. (**D**) Quantification of band density revealed no difference in KIM-1 protein expression between control and knockout injured kidneys. (**E**) qPCR was performed to analyze the expression of *Lcn2* message, the gene which encodes for NGAL. Data were analyzed by 2^−^^ΔΔ^^Ct^ method to determine fold change from the control, uninjured kidney. (**F**) qPCR data were again analyzed per individual mouse to assess *Lcn2* expression in the kidney after injury, relative to the uninjured kidney. (**G**) Western blot detected significant expression of NGAL only in the injured kidneys from both groups, with no significant difference between levels measured in injured kidneys from control and knockout animals (**H**). n = 12 to 16 for each measure; data represented as mean ± SD; unpaired t-tests or one-way ANOVA with multiple comparisons performed to determine significant differences with ** < 0.005, *** < 0.0005, **** < 0.00005.

Next, we examined whole kidney expression of the renal injury markers kidney injury marker-1 (KIM-1) and neutrophil gelatinase-associated lipocalin (NGAL) which indicate proximal and distal tubule damage respectively [22]. mRNA levels of *Havcr1*, the gene which encodes for KIM-1, were significantly increased in the injured versus uninjured kidneys of both groups of animals. The injured kidneys of knockout animals displayed a significantly higher fold change in *Havcr1* mRNA compared to the injured kidneys of control animals (Figure 3A). However, when examining the fold increase in *Havcr1* mRNA levels in injured versus uninjured kidney within each individual animal, no difference was noted between control and knockout groups (Figure 3B). At a protein level, KIM-1 was not detected in uninjured kidneys by Western blot (Figure 3C), and no significant difference was observed in levels of KIM-1 expression between the injured kidneys of control and knockout animals (Figure 3D).

Injured kidneys from both control and knockout animals displayed a significant increase in *Lcn2* mRNA levels, the gene that encodes for NGAL, with no significant difference between expression in injured kidneys from knockout and control animals (Figure 3E). Analysis of the fold increase in expression in *Lcn2* in injured versus uninjured kidney within each individual animal also revealed no significant difference between the control and knockout mice (Figure 3F). NGAL protein expression was again only detectable in injured tissue (Figure 3G) and was not significantly different between control and knockout animals (Figure 3H).

### 2.3. Histologic Analysis Reveals No Differences after IRI between Control and Knockout Animals

**Figure 4:**
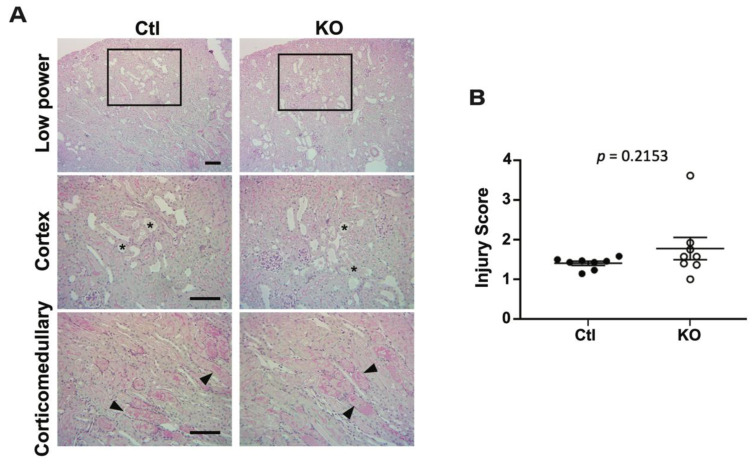
Histological examination reveals no difference in tubule injury. Kidney sections from control and knockout animals were stained with periodic acid-Schiff (PAS) to assess the extent of tubule injury with IRI. (**A**) Representative images from control and knockout injured kidneys reveal the presence of damaged tubules (top panels). The area indicated by the black box was enlarged (middle panels) to show the presence of dilated tubules with luminal casts (indicated by stars), and the corticomedullary region showed diffuse injury of tubules (indicated by arrowheads). (**B**) Sections were evaluated by an investigator blinded to animal group. No difference was observed in the extent of damage between injured kidneys from control and knockout animals, as analyzed by unpaired t-test. Uninjured kidneys were also analyzed, with no injury being observed in either genotype (images not shown). Bar denotes 100 µm.

To examine the structural changes that occurred in the kidneys with IRI, formalin fixed kidneys from control and knockout animals were stained with periodic acid-Schiff to detect the presence of polysaccharides. Uninjured kidneys showed no abnormal histology (not shown). However ischemia-reperfusion led to the presence of dilated tubules with luminal casts (Figure 4A, indicated by stars) and injured tubules (Figure 4A, indicated by arrows). Comparison of injury scores (Figure 4B) showed no significant difference in the extent of injury between control and knockout animals.

### 2.4. eNOS Phosphorylation Is Increased in Injured Kidneys of Knockout Animals

**Figure 5:**
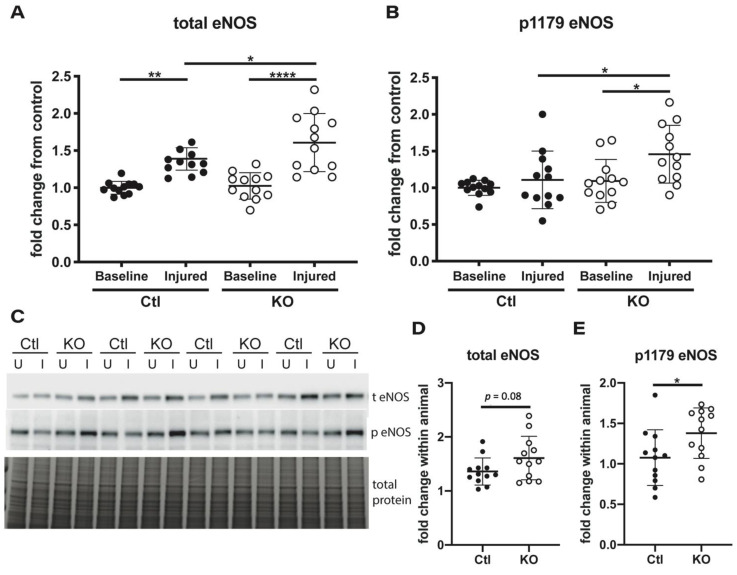
eNOS Phosphorylation at Ser1179 is increased in injured kidneys of endothelial γ ENaC knockout mice. Whole kidney lysates from uninjured (U) and injured (I) kidneys from control and knockout mice were assessed by SDS-page electrophoresis for levels of total eNOS and phosphorylated eNOS (p1179 eNOS). (**A**) Total eNOS was quantified and compared to levels in uninjured, control kidneys and expressed as fold-change from uninjured, control kidneys. (**B**) The same kidneys were examined for phosphorylation at Ser1179 (**C**) Representative blots for both total and phosphorylated eNOS are shown with total protein for normalization. The change in expression levels between the uninjured and injured kidney from each animal was calculated as a fold change for both total (**D**) and phosphorylated (**E**) eNOS. n = 12 to 16 for each measure; data presented as mean ± SD; unpaired *t*-tests or one-way ANOVA with multiple comparisons performed to determine significant differences with * < 0.05, ** < 0.005, **** < 0.00005.

Previous studies have shown that deletion or pharmacological inhibition of ENaC in endothelial cells increases NO production through modulation of cellular deformability that ultimately affects phosphorylation and activation of eNOS [23,24]. To investigate this in our model, we measured levels of total and phosphorylated eNOS at serine 1179 (corresponding to Ser1177 in human) by Western blot (Figure 5C) to understand if the enzyme is potentially more active in knockout animals. Total eNOS expression was significantly increased in the injured kidneys of both groups, compared to uninjured kidneys. However, the increase was significantly higher in the injured kidneys from knockout mice compared to controls (Figure 5A). The control animals had no difference in eNOS phosphorylation status between the uninjured and injured kidneys, however, the knockout animals displayed significantly higher phosphorylation of eNOS at serine 1179 in injured kidneys as compared to uninjured (Figure 5B). Analysis of the fold increase in total eNOS expression in injured versus uninjured kidney within each individual animal showed a slight, but not statistically significant increase between knockout and controls (Figure 5D, *p* = 0.08). However, a significant increase in phosphorylated eNOS was measured at the individual level (Figure 5E). These data indicate that a loss of **γ** ENaC expression leads to eNOS activation that could enhance NO production.

### 2.5. Immune Cell Infiltration and Cytokine Production Are Similar in Kidneys from Control and Knockout Animals

**Figure 6:**
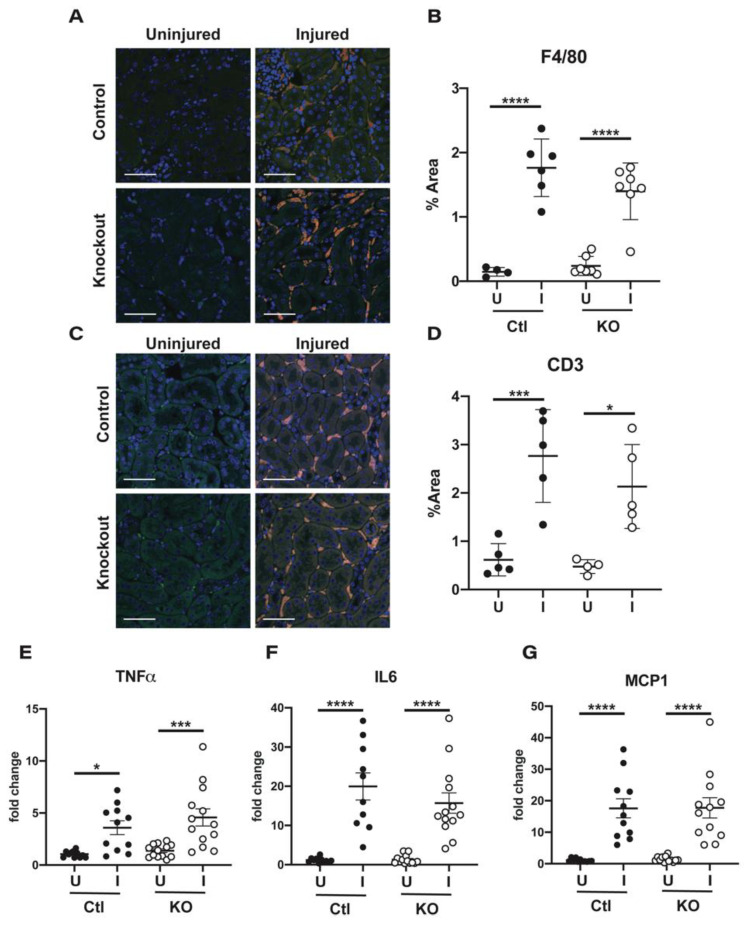
Immune cell infiltration and cytokine release do not differ between injured kidneys of control and endothelial γ ENaC knockout mice. Kidneys from control and knockout mice were examined for immune cell infiltration and cytokine production. (**A**) Sections of both uninjured and injured kidneys from control and knockout animals were labeled with an antibody to F4/80 (shown in red) to identify macrophages. In all images, green is autofluorescence of the kidney tissue to show the location of tubules and the blue channel denotes DAPI for nuclei identification. White bar denotes 50 μm. (**B**) The area within each section that stained positive for F4/80 was calculated as a percentage of the total area. (**C**) Kidneys were also labeled with an antibody against CD3 (shown in red) to identify T cells and (**D**) the images were quantified again as in B. Uninjured and injured kidneys were analyzed by qPCR for levels of mRNA encoding the pro-inflammatory cytokines TNFα (*Tnf*) (**E**), IL6 (*Il6*) (**F**), and MCP1 (*Ccl2)* (**G**). Data were analyzed by the 2^−^^ΔΔ^^Ct^ method to determine fold change from the control, uninjured kidney. For (**A**–**D**) n = 4 to 6 with data represented as mean ± SD. For (**E**–**G**) n = 10 to 13 with data presented as mean ± SEM. One-way ANOVA with multiple comparisons performed to determine significant differences with * < 0.05, *** < 0.0005, **** < 0.00005.

While canonical ENaC channels in the vasculature have been shown to play a role in promoting cellular stiffening to limit the activation of eNOS, the α subunit has also been shown to modulate vascular permeability [21,25]. This role is at least partially attributable to non-canonical channels containing the α subunit and ASIC1a [21]. Because the β and γ subunits are not known to associate with ASIC1a in endothelial cells, deletion of these subunits may lead to a different response if ASIC1a is necessary for α subunit mediated protection. To assess whether vascular permeability was altered in our γ subunit knockout animals, we looked at immune cell infiltration and pro-inflammatory cytokine production. Staining sections of both uninjured and injured kidneys from control and knockout animals with an antibody against F4/80 indicated that significant accumulation of macrophages only occurred under conditions of injury (Figure 6A). Two to three sections from each kidney were imaged at low magnification and quantified for the percentage of area that stained positive for the marker, with data from the different sections being averaged together for one point (Figure 6B). No significant difference in positive area was measured between injured kidneys from control and knockout animals. Levels of the T cell marker CD3 were also examined (Figure 6C), and again, no significant difference in positive-staining area was noted (Figure 6D), suggesting no difference in immune cell infiltration between the control and knockout animals in response to injury.

The pro-inflammatory cytokines TNF-α, IL-6, and MCP-1 were examined at a transcript level by qPCR (Figure 6E–G). While production was highly variable between animals, transcript levels were increased with injury with no significant difference in induction noted between control and knockout animals.

## 3. Discussion

Data presented here demonstrate that deletion of the γ subunit of ENaC in endothelial cells is not protective against renal IRI, as assessed by blood levels of BUN and creatinine, levels of kidney injury markers KIM-1 and NGAL, markers of inflammation, and histologic analyses. However, our data do suggest that deletion of this subunit is potentially associated with greater eNOS activation, as assessed by its phosphorylation at Ser1179 in injured kidneys in knockout versus control mice. These results were unexpected given the previously published work demonstrating that deletion of the α subunit is protective in renal IRI [16]. There are several possibilities for this discrepancy, discussed below.

Differences in the IRI model employed could explain the differential results. Experiments with the α subunit endothelial specific knockout mice used bilateral IRI for 22 min, with sacrifice and tissue collection at 24 h [18]. Our experiments used a unilateral IRI model in which an uninjured kidney was removed at the time of surgery. The remaining kidney was exposed to IRI for 20 min and tissue collected 48 h later. We selected the unilateral IRI approach to improve consistency of injury [26]. An additional benefit is the presence of a normal uninjured kidney as an internal control. We cannot completely eliminate the possibility that these small surgical differences could account for the discrepancy in our data compared to prior work, however, other factors may contribute.

Reperfusion time may also account for the differences in our results due to the multi-faceted role of NO in IRI. There are two well established sources of NO in the kidney: (i) endothelial cells which produce NO through eNOS and (ii) immune cells that contribute NO through inducible nitric oxide synthase (iNOS) [27]. While the former is considered to be protective in IRI due to its ability to cause vasodilation, the latter has been shown to promote peroxynitrite formation and inflammation, enhancing kidney damage [28,29]. These opposing effects mediated by the same molecule demonstrate the importance of source and timing of NO production in determination of its downstream effects in the setting of IRI, and show that NO can turn from being helpful to detrimental.

Levels of eNOS increase acutely in kidneys subjected to IRI, which presumably facilitates vasodilation and an increase in blood flow to ischemic tissue to restore oxygenation [7]. After 48 h of reperfusion, we found that total eNOS levels were elevated in the injured kidneys from both control and endothelial γ ENaC knockout mice. However, the increase in eNOS was significantly greater in knockout mice. Further, the activating phosphorylation of eNOS at Ser1179 was significantly higher in the knockout injured kidney compared to both the uninjured kidney in knockout mice and the injured and uninjured kidneys of control mice. These data suggest that eNOS activation is significantly greater in injured kidneys in knockout mice. As superoxide levels increase with reperfusion, higher production of NO may be detrimental over time, given its ability to react with superoxide to form peroxynitrite. This potent reactive oxygen species has been shown to be detrimental in IRI, as it impairs mitochondrial function, increases inflammation, and disrupts DNA structure [30]. Furthermore, genetic deletion of eNOS has been demonstrated to limit renal injury in the setting of ischemia-reperfusion, as assessed by lower blood creatinine levels, immune cell infiltration and nitrotyrosine staining of injured kidneys in eNOS knockout mice [7]. This may occur because genetic deletion of eNOS may prevent the activation of iNOS by eNOS-derived NO, limiting the potential for inflammation [31]. However, other vasodilators would potentially have to compensate in these animals, to ensure that proper reoxygenation occurred.

It is also possible that the α subunit of ENaC may form non-canonical ENaC/degenerin channels in endothelial cells. Work in pulmonary endothelium has demonstrated the existence of hybrid ENaC/ASIC formed by the α subunit of ENaC and ASIC1a. Similar interactions between these two proteins have been described in alveolar [32] and cancer cells [33,34]. Furthermore, ENaC channels consisting of only one or two subunits (i.e., α subunit alone, or α,β channels) have limited activity. Genetic deletion of the γ subunit of ENaC in endothelia would not affect these non-canonical ENaC/degenerin channels. Much of the work elucidating the role of ENaC in endothelial function has utilized α subunit knockout mice or ENaC inhibitors amiloride or benzamil [16,18,21,24,25,35,36]. These inhibitors would also block non-canonical ENaC/degenerin channels, albeit with differing kinetics [34]. Because of the limited expression of ENaC in the vascular endothelium, expression analysis has proven difficult in our hands. Further analysis is needed to understand if ENaC subunit expression differs across different vascular beds or in the settings of hypoxia, aging, or increased aldosterone levels.

Together, our data demonstrate that deletion of the γ subunit of ENaC in endothelial cells does not protect against renal IRI, in spite of higher total levels of eNOS with increased eNOS Ser1179 phosphorylation. One caveat of our work is that we did not assess NO levels, as accurate measurement of such a short-lived molecule is highly variable and challenging in tissues. Regardless, our negative results suggest that the phenotype of these animals differs from that of an α subunit endothelial specific knockout mouse, suggesting distinct roles for these subunits. Further work is necessary to better understand the contributions of individual ENaC subunits in normal endothelia and in disease states.

## 4. Materials and Methods

### 4.1. Animals

#### Generation of γ ENaC Floxed Mice

Based on the γ ENaC gene (*Scnn1g*), a targeting construct was made containing 3 kb of flanking homologous DNA, a flippase recognition target–flanked (FRT-flanked) PGK promoter-NeoR sequence, and 2 loxP sites surrounding exons 2 and 3, encoding the N-terminus, first transmembrane domain and part of the extracellular domain. Deletion of this region is predicted to cause a complete loss of function [37]. Thymidine kinase sequences were placed on either end of the vector.

Mouse SVJ129 embryonic stem cells (from the Transgenic Core at the University of Utah) were electroporated with the vector. Several γ ENaC ES clones grown under neomycin and thymidine analogue selection were screened by PCR analysis, and their identity confirmed by Southern analysis. One clone with a normal karyotype was identified, and this was used to inject into recipient C57BL/6J blastocysts. Founder agouti mice were obtained from the Jackson Laboratory (Bar Harbor, ME, USA), bred to C57BL/6J mice, and assessed for germline transmission of the floxed allele by tail vein clipping and genotyping. These mice were then bred with mice expressing Flp recombinase to excise the FRT-flanked PGK promoter-NeoR cassette. Pups were analyzed for excision of the cassette, and the desired genotype was bred with C57BL/6J WT mice to eliminate Flp recombinase. 

### 4.2. IRI Surgery

Homozygous floxed mice were crossed to homozygous floxed mice also expressing cre under control of the Tie2 promoter (Jackson Labs, Bar Harbor, ME, USA) to generate an endothelial specific γ ENaC knockout. Floxed animals not expressing cre were used as littermate controls. Male mice, aged 8–12 weeks, were subjected to unilateral IRI with simultaneous contralateral nephrectomy as previously described [26]. Briefly, mice were anesthetized and a laparotomy performed. The left kidney was isolated and the renal pedicle clamped with atraumatic surgical clips (RS-5459; Roboz, Gaithersburg, MD, USA) for 20 min. During this period, the right kidney was exposed, and the renal artery and vein were occluded with silk suture. This kidney was excised and used as uninjured tissue for analyses. The clamp was removed, the kidney allowed to reperfuse, and the incision closed. Mice were euthanized for final analysis after 48 h of reperfusion at which time blood was collected by retroorbital bleed and serum separated by centrifugation, and the injured kidney was divided and frozen in liquid nitrogen or fixed in formalin.

All animal protocols were approved by the Institutional Animal Care and Use Committee at the University of Pittsburgh (PHS approval number D16-0018). A total of 35 animals were used for this study, on protocol #19075553, initially approved 7/12/2019.

### 4.3. Measure of Renal Dysfunction and Injury

Serum was collected at 48 h of reperfusion at the time of animal sacrifice. Commercially available kits were utilized to measure urea (blood urea nitrogen, BioAssay Systems, Hayward, CA, USA) and creatinine levels (Pointe Scientific, Canton, MI, USA) according to the manufacturer’s instructions. Histology was examined in both the uninjured kidney excised at the time of IRI surgery and the injured kidney excised at the time of sacrifice. The tissue was fixed in 10% neutral buffered formalin and embedded in paraffin, sectioned, and stained with a standard periodic acid-Schiff (PAS) protocol. To score tissue injury, high power microscopic images comprising the entire cortex and corticomedullary region of a single tissue section for each mouse were obtained. The images were scored by an investigator blinded to group assignment according to the following scale: 0 = no injury; 1 = 1 to 25% of parenchyma affected by injury; 2 = 26–50% involvement; 3 = 51–75% involvement; and 4 = 76–100% involvement. Injury was defined as dilated tubules, casts, cell sloughing, or loss of brush borders. Scores for the images from each mouse were averaged to obtain a single score for that animal, and then a group average was calculated.

### 4.4. Quantitative, Real-Time, Reverse-Transcriptase Polymerase Chain Reaction

Whole kidney mRNA was isolated using Trizol (Invitrogen, Waltham, MA, USA). One microgram of total RNA was added to a cDNA synthesis kit (Bio-Rad Laboratories, Hercules, CA, USA) and cDNA was added to reactions containing SYBR green master mix (Bio-Rad Laboratories, Hercules, CA, USA) and a pairing of the following primers: *Havcr1* forward 5′-TTC AGG AAG CTG AGC AAA CAT-3′, *Havcr1* reverse 5′-CCCCAACATGTCGTTGTGATT-3′, *Lcn2* forward 5′-CCATCTATGAGCTACAAGAGAACAAT-3′, *Lcn2* reverse 5′-TCTGATCCAGTAGCGACAGC-3′, *Tnf* forward 5′-TCGTAGCAAACCACCAAGTG-3′, *Tnf* reverse 5′-CCTTGAAGAGAACCTGGGAG-3′, *Il6* forward 5′-CTTGGGACTGATGCTGGTG-3′, *Il6* reverse 5′-TCCACGATTTCCCAGAGAAC-3′, *Ccl2* forward 5′-CCCACTCACCTGCTGCTA-3′, *Ccl2* reverse 5′-TTCTTGGGGTCAGCACAGA-3′, *18S* forward 5′-GTAACCCGTTGAACCCCATT-3′, *18S* reverse 5′-CCATCCAATCGGTAGTAGCG-3′. Differences in expression were calculated using the 2^−^^ΔΔ^^Ct^ method.

### 4.5. SDS Page Electrophoresis

Snap-frozen kidney pieces were homogenized in CellLytic^TM^ MT Cell Lysis reagent (Sigma-Aldrich, St. Louis, MO, USA) supplemented with Halt protease and phosphatase inhibitor cocktail (Thermo Fisher, Waltham, MA, USA). Protein concentration was determined with a BCA protein assay kit (Thermo Scientific, Rockford, IL, USA). 35 µg of homogenized protein were prepared/well in 2x Laemmli buffer (Bio-Rad laboratories, Hercules, CA, USA). Tissue lysates were run on a 4–15% Criterion TGX precast gel (Bio-Rad Laboratories). Membranes were incubated with primary antibody overnight at 4 °C as follows: KIM-1 (1:1500 or 0.13 µg/mL, R&D Systems, Minneapolis, MN, USA), NGAL (1:1500 or 0.13 µg/mL, R&D Systems, Minneapolis, MN, USA), total eNOS (1:1000 or 0.25 µg/mL, BD Biosciences, Franklin Lakes, NJ, USA), p1177 eNOS (1:1000 or 0.25 µg/mL, BD Biosciences, Franklin Lakes, NJ). Secondary antibodies were applied the next day for 1 h at room temperature (1:10,000 KPLaboratories, Gaithersburg, MD, USA) and blots were developed using Clarity™ Western Blotting Substrate (Bio-Rad Laboratories). A ChemiDoc™ Touch Imaging System was used for exposure (Bio-Rad Laboratories). Densitometry was performed in Image J software [38], and bands of interest were normalized to total protein as determined by a simultaneously run Coomassie stained gel.

### 4.6. Immunofluorescent Analysis

Sections of kidney tissue were deparaffinized and antigen retrieval performed in citrate buffer. Tissue was incubated overnight at 4 °C in primary antibody directed against CD3 (1:100, R&D Systems, Minneapolis, MN, USA) or F4/80 (1:100, Bio-Rad Laboratories, Hercules, CA, USA). Fluorescently labeled secondary antibodies (Jackson ImmunoResearch, West Grove, PA, USA) were applied the following day at room temperature for one hour, nuclei were stained with DAPI, and coverslips were mounted using ProLong Gold Antifade Mountant (Thermo Fisher, Waltham, MA, USA). Image stacks were taken on a Fluoview-1000 microscope with two to three regions examined per kidney. ImageJ [38] was used to threshold the channel of interest and the positive stained area was calculated and averaged across the images.

### 4.7. Statistical Analysis

Data were analyzed using GraphPad Prism software [39] and are presented as means  ±  SD. All comparisons between four groups were determined using one-way ANOVA followed by the appropriate post hoc test for individual comparisons. Student’s *t*-test was used for two groups. Outliers were tested for and excluded using Grubbs test. Significance was assumed to be *p* ≤ 0.05.

## Data Availability

The data analyzed for this study are contained, in whole, within the manuscript.

## References

[1] Han S.J., Lee H.T. (2019). Mechanisms and therapeutic targets of ischemic acute kidney injury. Kidney Res. Clin. Pract..

[2] Nourbakhsh N., Singh P. (2014). Role of renal oxygenation and mitochondrial function in the pathophysiology of acute kidney injury. Nephron Clin. Pract..

[3] Wu M.Y., Yiang G.T., Liao W.T., Tsai A.P., Cheng Y.L., Cheng P.W., Li C.Y., Li C.J. (2018). Current Mechanistic Concepts in Ischemia and Reperfusion Injury. Cell Physiol. Biochem..

[4] Basile D.P., Yoder M.C. (2014). Renal endothelial dysfunction in acute kidney ischemia reperfusion injury. Cardiovasc Hematol. Disord Drug Targets.

[5] Saito M., Miyagawa I. (2000). Real-time monitoring of nitric oxide in ischemia-reperfusion rat kidney. Urol. Res..

[6] Katsumi H., Takashima R., Suzuki H., Hirai N., Matsuura S., Kimura H., Morishita M., Yamamoto A. (2020). S-nitrosylated l-serine-modified dendrimer as a kidney-targeting nitric oxide donor for prevention of renal ischaemia/reperfusion injury. Free Radic. Res..

[7] Milsom A.B., Patel N.S., Mazzon E., Tripatara P., Storey A., Mota-Filipe H., Sepodes B., Webb A.J., Cuzzocrea S., Hobbs A.J. (2010). Role for endothelial nitric oxide synthase in nitrite-induced protection against renal ischemia-reperfusion injury in mice. Nitric Oxide.

[8] Yang T., Zhang X.M., Tarnawski L., Peleli M., Zhuge Z., Terrando N., Harris R.A., Olofsson P.S., Larsson E., Persson A.E.G. (2017). Dietary nitrate attenuates renal ischemia-reperfusion injuries by modulation of immune responses and reduction of oxidative stress. Redox Biol..

[9] Mutchler S.M., Straub A.C. (2015). Compartmentalized nitric oxide signaling in the resistance vasculature. Nitric Oxide.

[10] Fleming I. (2010). Molecular mechanisms underlying the activation of eNOS. Pflugers Arch..

[11] Ghimire K., Zaric J., Alday-Parejo B., Seebach J., Bousquenaud M., Stalin J., Bieler G., Schnittler H.J., Ruegg C. (2019). MAGI1 Mediates eNOS Activation and NO Production in Endothelial Cells in Response to Fluid Shear Stress. Cells.

[12] Zhang Y., Lee T.S., Kolb E.M., Sun K., Lu X., Sladek F.M., Kassab G.S., Garland T., Shyy J.Y. (2006). AMP-activated protein kinase is involved in endothelial NO synthase activation in response to shear stress. Arterioscler. Thromb. Vasc. Biol..

[13] Fels J., Callies C., Kusche-Vihrog K., Oberleithner H. (2010). Nitric oxide release follows endothelial nanomechanics and not vice versa. Pflugers Arch..

[14] Mutchler S.M., Kirabo A., Kleyman T.R. (2021). Epithelial Sodium Channel and Salt-Sensitive Hypertension. Hypertension.

[15] Jeggle P., Callies C., Tarjus A., Fassot C., Fels J., Oberleithner H., Jaisser F., Kusche-Vihrog K. (2013). Epithelial sodium channel stiffens the vascular endothelium in vitro and in Liddle mice. Hypertension.

[16] Jia G., Habibi J., Aroor A.R., Hill M.A., Yang Y., Whaley-Connell A., Jaisser F., Sowers J.R. (2018). Epithelial Sodium Channel in Aldosterone-Induced Endothelium Stiffness and Aortic Dysfunction. Hypertension.

[17] Kusche-Vihrog K., Tarjus A., Fels J., Jaisser F. (2014). The epithelial Na+ channel: A new player in the vasculature. Curr. Opin. Nephrol. Hypertens.

[18] Tarjus A., Gonzalez-Rivas C., Amador-Martinez I., Bonnard B., Lopez-Marure R., Jaisser F., Barrera-Chimal J. (2019). The Absence of Endothelial Sodium Channel alpha (alphaENaC) Reduces Renal Ischemia/Reperfusion Injury. Int. J. Mol. Sci..

[19] Kashlan O.B., Kleyman T.R. (2011). ENaC structure and function in the wake of a resolved structure of a family member. Am. J. Physiol. Renal. Physiol..

[20] Jeggle P., Smith E.S., Stewart A.P., Haerteis S., Korbmacher C., Edwardson J.M. (2015). Atomic force microscopy imaging reveals the formation of ASIC/ENaC cross-clade ion channels. Biochem. Biophys. Res. Commun..

[21] Czikora I., Alli A.A., Sridhar S., Matthay M.A., Pillich H., Hudel M., Berisha B., Gorshkov B., Romero M.J., Gonzales J. (2017). Epithelial Sodium Channel-alpha Mediates the Protective Effect of the TNF-Derived TIP Peptide in Pneumolysin-Induced Endothelial Barrier Dysfunction. Front. Immunol..

[22] Westhoff J.H., Seibert F.S., Waldherr S., Bauer F., Tonshoff B., Fichtner A., Westhoff T.H. (2017). Urinary calprotectin, kidney injury molecule-1, and neutrophil gelatinase-associated lipocalin for the prediction of adverse outcome in pediatric acute kidney injury. Eur. J. Pediatr.

[23] Fels J., Oberleithner H., Kusche-Vihrog K. (2010). Menage a trois: Aldosterone, sodium and nitric oxide in vascular endothelium. Biochim. Biophys. Acta.

[24] Perez F.R., Venegas F., Gonzalez M., Andres S., Vallejos C., Riquelme G., Sierralta J., Michea L. (2009). Endothelial epithelial sodium channel inhibition activates endothelial nitric oxide synthase via phosphoinositide 3-kinase/Akt in small-diameter mesenteric arteries. Hypertension.

[25] Sternak M., Bar A., Adamski M.G., Mohaissen T., Marczyk B., Kieronska A., Stojak M., Kus K., Tarjus A., Jaisser F. (2018). The Deletion of Endothelial Sodium Channel alpha (alphaENaC) Impairs Endothelium-Dependent Vasodilation and Endothelial Barrier Integrity in Endotoxemia in Vivo. Front. Pharmacol..

[26] Skrypnyk N.I., Harris R.C., de Caestecker M.P. (2013). Ischemia-reperfusion model of acute kidney injury and post injury fibrosis in mice. J. Vis. Exp..

[27] Sedaghat Z., Kadkhodaee M., Seifi B., Salehi E. (2019). Inducible and endothelial nitric oxide synthase distribution and expression with hind limb per-conditioning of the rat kidney. Arch. Med. Sci..

[28] Chatterjee P.K., Patel N.S., Kvale E.O., Cuzzocrea S., Brown P.A., Stewart K.N., Mota-Filipe H., Thiemermann C. (2002). Inhibition of inducible nitric oxide synthase reduces renal ischemia/reperfusion injury. Kidney Int..

[29] Chen C., Sun L., Zhang W., Tang Y., Li X., Jing R., Liu T. (2020). Limb ischemic preconditioning ameliorates renal microcirculation through activation of PI3K/Akt/eNOS signaling pathway after acute kidney injury. Eur. J. Med. Res..

[30] Soliman E., Shewaikh S.M., Fahmy A., Elshazly S. (2021). Entacapone scavenges peroxynitrite and protects against kidney and liver injuries induced by renal ischemia/reperfusion in rats. Int. Urol. Nephrol..

[31] Vo P.A., Lad B., Tomlinson J.A., Francis S., Ahluwalia A. (2005). autoregulatory role of endothelium-derived nitric oxide (NO) on Lipopolysaccharide-induced vascular inducible NO synthase expression and function. J. Biol. Chem..

[32] Trac P.T., Thai T.L., Linck V., Zou L., Greenlee M., Yue Q., Al-Khalili O., Alli A.A., Eaton A.F., Eaton D.C. (2017). Alveolar nonselective channels are ASIC1a/alpha-ENaC channels and contribute to AFC. Am. J. Physiol. Lung Cell Mol. Physiol..

[33] Kapoor N., Bartoszewski R., Qadri Y.J., Bebok Z., Bubien J.K., Fuller C.M., Benos D.J. (2009). Knockdown of ASIC1 and epithelial sodium channel subunits inhibits glioblastoma whole cell current and cell migration. J. Biol. Chem..

[34] Kapoor N., Lee W., Clark E., Bartoszewski R., McNicholas C.M., Latham C.B., Bebok Z., Parpura V., Fuller C.M., Palmer C.A. (2011). Interaction of ASIC1 and ENaC subunits in human glioma cells and rat astrocytes. Am. J. Physiol. Cell Physiol..

[35] Martinez-Lemus L.A., Aroor A.R., Ramirez-Perez F.I., Jia G., Habibi J., DeMarco V.G., Barron B., Whaley-Connell A., Nistala R., Sowers J.R. (2017). Amiloride Improves Endothelial Function and Reduces Vascular Stiffness in Female Mice Fed a Western Diet. Front. Physiol..

[36] Sowers J.R., Habibi J., Aroor A.R., Yang Y., Lastra G., Hill M.A., Whaley-Connell A., Jaisser F., Jia G. (2019). Epithelial sodium channels in endothelial cells mediate diet-induced endothelium stiffness and impaired vascular relaxation in obese female mice. Metabolism.

[37] Bruns J.B., Hu B., Ahn Y.J., Sheng S., Hughey R.P., Kleyman T.R. (2003). Multiple epithelial Na+ channel domains participate in subunit assembly. Am. J. Physiol. Renal. Physiol..

[38] Rasband W.S. (2011). ImageJ, U.S. National Institutes of Health, Bethesda, Maryland, USA. https://www.semanticscholar.org/paper/ImageJ%2C-U.S.-National-Institutes-of-Health%2C-USA-Rasband/034dbc2e4c735500c519183180f8cf6033fcb28d.

[39] (2021). GraphPad Prism.

